# A New Method for Training CycleGAN to Enhance Images of Cold Seeps in the Qiongdongnan Sea

**DOI:** 10.3390/s23031741

**Published:** 2023-02-03

**Authors:** Yuanheng Li, Shengxiong Yang, Yuehua Gong, Jingya Cao, Guang Hu, Yutian Deng, Dongmei Tian, Junming Zhou

**Affiliations:** 1 Southern Marine Science and Engineering Guangdong Laboratory (Guangzhou), Guangzhou 511458, China; 2 Ocean College and Institute of Ocean Research, Fujian Polytechnic Normal University, Fuzhou 350300, China; 3 Guangzhou Marine Geological Survey, Guangzhou 510075, China

**Keywords:** cold seeps, underwater image enhancement, CycleGAN, MSRCR

## Abstract

Clear underwater images can help researchers detect cold seeps, gas hydrates, and biological resources. However, the quality of these images suffers from nonuniform lighting, a limited range of visibility, and unwanted signals. CycleGAN has been broadly studied in regard to underwater image enhancement, but it is difficult to apply the model for the further detection of Haima cold seeps in the South China Sea because the model can be difficult to train if the dataset used is not appropriate. In this article, we devise a new method of building a dataset using MSRCR and choose the best images based on the widely used UIQM scheme to build the dataset. The experimental results show that a good CycleGAN could be trained with the dataset using the proposed method. The model has good potential for applications in detecting the Haima cold seeps and can be applied to other cold seeps, such as the cold seeps in the North Sea. We conclude that the method used for building the dataset can be applied to train CycleGAN when enhancing images from cold seeps.

## 1. Introduction

Exploring cold seeps in the deep water of the South China Sea has attracted extensive scientific interest [1]. Current research reveals that the escape of submarine cold seeps often reflects the existence of methane hydrates or deep natural gas [2]. Feng et al. conducted a quantitative study on the contribution of cold seep fluids to the bottom-water carbon reservoir of the cold seeps in the South China Sea. These cold seeps might help our understanding of the dynamics and the environmental impacts of hydrocarbons [3]. Di et al. determined size distribution, bubble release rate, and bubble diameters using a semiautomatic bubble-counting algorithm and observed that the Haima cold seeps in the South China Sea may be a source of methane for the ocean [4].

Clear underwater images can be a powerful tool for researchers in detecting cold seeps, gas hydrates, and biological resources. However, complex illumination conditions in the water, such as nonuniform lighting, diminished colors, and unwanted signals, limit the range of optical detection [5]. To solve these problems, underwater image enhancement technology has been widely studied [6]. The white balance method and the shades-of-gray method have been successfully applied [7,8,9]. However, at the same time, these methods may lead to image distortion because of the overcompensation of the red channel [10]. Those methods do not consider the fact that the color perceived by the retina is determined by an object’s ability to reflect long-, medium-, and short-wavelength light. Thus, they always suffer from an imbalance with respect to color channels. In consideration of the issues mentioned above, a visual model named retinex was created to explain perceived human color [11,12,13]. Joshi and Kamathe applied retinex to underwater image enhancement [14]. With retinex processing, one can obtain a dynamic range or color and lightness rendition compression, but these cannot be obtained simultaneously. Therefore, Rahman et al. presented a multi-scale retinex model to overcome this limitation [15]. Furthermore, Jobson et al. proposed a multi-scale retinex model with color restoration (MSRCR) to solve the distortion defect problem caused by strong local contrasts [16]. With the development of retinex, MSRCR has also been applied to underwater image enhancements, and good results have been achieved [17]. However, the underwater environment is complex, and MSRCR is not flexible enough; thus, we need to debug the results many times in order to obtain good enhancement results to improve its application range [6]. To solve this problem, Hu et al. introduced the natural image quality evaluation index and a gravitational search algorithm in a multi-scale retinex model to obtain a parameter index with an excellent adaptive ability relative to environmental changes [18].

With the development of artificial intelligence (AI), underwater image enhancements based on AI have achieved good results [19]. Perez et al. [20] proposed an underwater image enhancement method using a convolutional neural network (CNN) that can be regarded as the first application of the deep learning method to underwater image enhancement. Anwar et al. later enhanced underwater images by constructing an underwater CNN [21]. Although both CNNs and underwater CNNs have been successfully applied in the field of underwater image enhancement, the complexity and scarcity of underwater images seriously restrict their applications [22]. Using deep learning frameworks can effectively improve the enhancement’s quality, although they fail to preserve the details of far-off objects in underwater images. To overcome these issues, Han et al. proposed a deep supervised residual dense net. It first uses residual dense blocks to extract features, and then the net adds residual path blocks between the encoder and decoder to reduce semantic differences between low-level features and high-level features; thus, it achieves good qualitative and quantitative enhancement effects [23]. Hu et al. introduced the natural image quality evaluation index to provide generated images with higher contrasts. The enhanced image of this algorithm is clearer than the truth image set provided by the existing dataset [24]. Zhang et al. used a two-stage detection network and a self-built dataset to construct an end-to-end model and improved the detection speed by 6% [25].

Generative adversarial nets (GANs) provide another possibility for transferring an image from one domain to another [26]. Domain conversion can be performed by using CycleGAN in the absence of paired data [27], thereby providing a new idea for underwater image enhancement. Fabbri et al. proposed UGAN, which uses CycleGAN to generate degraded underwater image data and improve the image quality [28]. A weak model inspired by CycleGAN was proposed by Goodfellow et al. [26]; it uses structural loss rather than the consistency loss of CycleGAN to generate a repaired image from the distorted image. This method addresses the influence of different types of water on the enhancement results, but it often produces strange textures and is highly dependent on the training dataset. Ahn et al. [29] used matched clear and unclear underwater images as a dataset for training, and the model structure of CycleGAN was adjusted to improve the effect. The multichannel CycleGAN technology proposed by Lu et al. [30] addresses the influence of different types of water on the enhancement results, but it is also highly dependent on the training dataset. An end-to-end underwater image enhancement method based on CycleGAN for an unpaired dataset was proposed by Du et al. in 2022. They used URPC2019 and EUVP datasets to train the model, effectively restored the blue-green background, and transformed a blurred underwater degraded image into a clear image [31]. Their work proves that CycleGAN can effectively enhance underwater images, but because its dataset requires clear underwater images and fuzzy underwater images at the same time, training the dataset is often difficult in practical work; thus, the application of CycleGAN is limited [5]. In the process of deep-sea cold seep exploration, obtaining large, clear datasets for training is difficult due to the limitation of capital costs. Moreover, because CycleGAN can render photographs into their respective styles [27], when a large number of underwater images are required to train other AI models, the trained CycleGAN can also be used to generate photographs with the style of underwater images. CycleGAN plays a basic role in unpaired dataset training, but cycle consistency limits the training efficiency. To solve this issue, Jiang et al. proposed a new GAN named EnlightenGAN [32]. They proposed an effective and efficient GAN suitable for unpaired training and applied it to low-light image enhancement, and it easily enhanced real-world low-light images from different domains.

**Figure 1:**
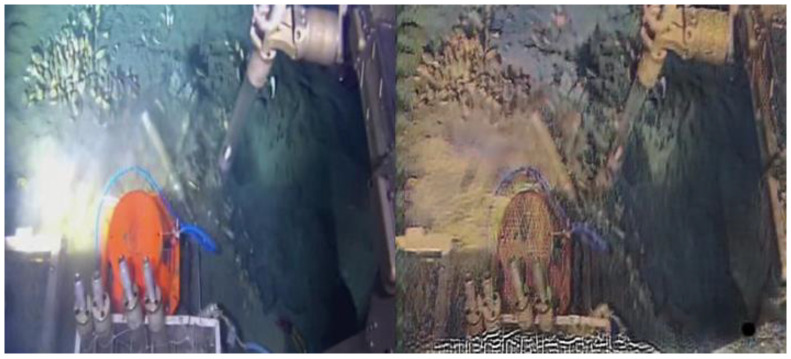
CycleGAN enhancement of an underwater image with an inappropriate dataset.

Although the method based on MSRCR has been widely used, it suffers from difficulties in parameter setting, limiting its practical application. Although the underwater image enhancement method based on CNNs has a good effect, it is difficult to apply because of the complex underwater environment it is expected to operate in and scarcity of data resources. CycleGAN provides a novel scheme for solving these problems, but training the network is difficult, especially an inappropriate land dataset is used, and the training effect of GAN may be elusive. Unfortunately, in the process of deep-sea cold seep exploration, it is often difficult to obtain a large number of clear images for training. Moreover, because CycleGAN can render photographs into their respective styles [27], when a large number of underwater images are required to train other AI models, the trained CycleGAN can also be used to generate photographs with the style of underwater images. As shown in Figure 1, when the dataset is not appropriate, it is difficult for CycleGAN to obtain good results via training. More effort needs to be directed toward finding a suitable method that can make CycleGAN practical for applications in cold seep underwater image enhancement.

When we used the standard model for training, we observed that it could not correctly enhance cold spring images from all environments. When we used the self-built dataset, if the dataset was inappropriate, we were not able to complete the model training. In order to solve these issues, we propose a new method of building datasets with the help of MSRCR and underwater image quality measurement (UIQM) to achieve good results. We used an actual image collected by a remotely operated vehicle (ROV) in a cold seep in Haima. Then, we chose images of the representative working conditions of the Haima ROV to test the enhancement effect of MSRCR with different parameters. After an automatic comparison with UIQM, the MSRCR parameters with the best effect were used to build the dataset to train CycleGAN. After hundreds of epochs, the model was trained for practical applications. The enhancement experiment using the trained CycleGAN demonstrated that the images were enhanced very well, and CycleGAN had a good generalization ability. Considering the above works, the main contributions of this paper are as follows:

- The effect of cold seep image enhancements using MSRCR on different imaging devices in different detectors was tested, and it was shown that a single enhancement coefficient or a fixed table could not meet the requirements of different scenes.
- CycleGAN was trained using the standard dataset and applied to the image enhancement of cold seep images. It was observed that the model worked well in some conditions and failed in other conditions.
- We found an effective way to build datasets to train CycleGAN with the help of MSRCR for cases in which a clear image dataset is difficult to obtain.
- Finally, an active underwater image enhancement CycleGAN that can be applied to practical applications rather than standard data models was trained. Compared with previous studies, the training ideas proposed in this paper may be applied to any underwater scene, with good universal applicability.

The rest of this paper is organized as follows. The basic methods of MSRCR and CycleGAN are presented in Section 2 briefly. Section 3 provides the details of the tests and results of the experiments in this paper. A discussion and conclusions are presented in Section 4 and Section 5, respectively.

## 2. Materials and Methods

### 2.1. Principle of MSRCR

The word retinex is a combination of retina and cortex [11]. Retinex theory includes two aspects: an object is determined by the reflection ability of the object rather than the absolute value and the reflected light intensity, and the color of the object is not affected by the uniformity of illumination [16]. Ma et al. [33] systematically introduced the idea of underwater image enhancement using MSRCR. Although MSRCR can yield good results when applied to underwater image enhancement, it is often necessary to test and update relevant parameters according to underwater illumination and turbidity conditions to obtain good image enhancement and color restoration, which limits its application [33].

### 2.2. Principle of CycleGAN

#### 2.2.1. Net Structure

**Figure 2:**
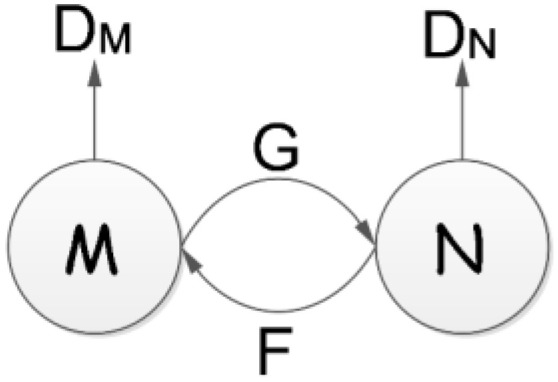
Basic structure of CycleGAN.

As shown in Figure 2, CycleGAN has four basic networks: two generators and two discriminators [27]. The generator network is used to generate two distributions, m and n. Generators G and F generate m domain data to n and n domain data to m, respectively. Their purpose is to generate images to deceive the two discriminator networks. Discriminators $D_{M}^{}$ and $D_{N}^{}$ judge the converted images and verify whether the image generated by *G* is *M* or *N*. A six-layer ResNet [34] is used to preserve the properties of the original input in the CycleGAN. Then as in [35], the discriminator network takes an image as input and tries to predict it as the original image or the output image of the generator.

#### 2.2.2. Cycle Consistency Loss 

**Figure 3:**
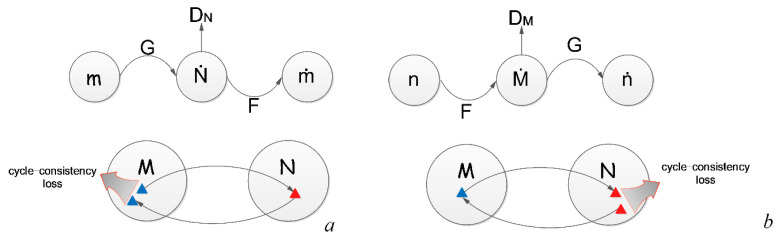
(**a**) Forward and (**b**) backward consistency losses.

We followed the design of reference [26] to calculate the loss of CycleGAN. As shown in Figure 3, *G*(*m*) can be obtained by substituting data *m* into generator *G*; by substituting *G*(*m*) into the inverse generator *F*, we can obtain *F*(*G*(*m*)) ≈ *m*, and the forward consistency loss can be obtained by calculating the loss, *L*. Similarly, we can obtain the backward consistency loss by calculating *G*(*F*(*n*)) ≈ *n*.

The related formula to calculate the cycle consistency loss $L_{cyc}^{}$ can be expressed as

(1) $$L_{cyc}^{}\left(G,F\right)=Em\sim p_{data}^{}{}_{\left(m\right)}^{}\left[{\left\|F\left(G\left(m\right)\right)-m\right\|}_{1}^{}\right]+Ey\sim p_{data}^{}{}_{\left(n\right)}^{}\left[{\left\|G\left(F\left(n\right)\right)-n\right\|}_{1}^{}\right],$$

where **E** indicates the calculation of the excepted value, and *p* is the probability. *F, G, m,* and *n* have the same meaning, as shown in Figure 3. Moreover, ‖ ‖ is the L1 norm. The full objective loss is

(2) $$L\left(G,F,Dm,Dn\right)=L_{GAN}^{}\left(G,Dn,X,Y\right)+L_{GAN}^{}\left(F,Dm,X,Y\right)+\lambda L_{cyc}^{}\left(G,F\right),$$

where *λ* is a permanent control of the relative importance of the two objectives. For the two sets to generate countermeasures, the network is reversed to solve

(3) $$G_{}^{*},F_{}^{*}=\arg\underset{G,F}{\min}\underset{Dm,Dn}{\max}\left(G,F,Dm,Dn\right).$$

## 3. Tests and Results

### 3.1. Dataset Preparation Using the MSRCR Method

Because the original MSRCR has so many parameters that make its application difficult, in this study, we selected the simplified MSRCR method in the GNU image manipulation program, which reduces the number of needed key parameters to one [36]. The method first calculates the mean value Mean and mean square deviation Var of each RGB channel of image $R\left(x,y\right)$. Then, the minimum and maximum values, Min and Max, of each channel are calculated using the following formulas:

(4) $$\left\{\begin{array}{l} Min=Mean-Dyn\cdot Var,\\ Max=Mean+Dyn\cdot Var, \end{array}\right.$$

where Dyn is the parameter used to adjust color saturation contamination around the new average color. This is the parameter for tweaking optimal results, because its effect is extremely image-dependent. Finally, the method performs linear mapping to output the enhanced image $\hat{R}(x,y)$ using the following formula:

(5) $$\hat{R}\left(x,y\right)=\left(\left(\;Value-Min\;\right)/\left(Max-Min\right)\right)\cdot 255,$$

where Value indicates the RGB value of each pixel in the image.

**Figure 4:**
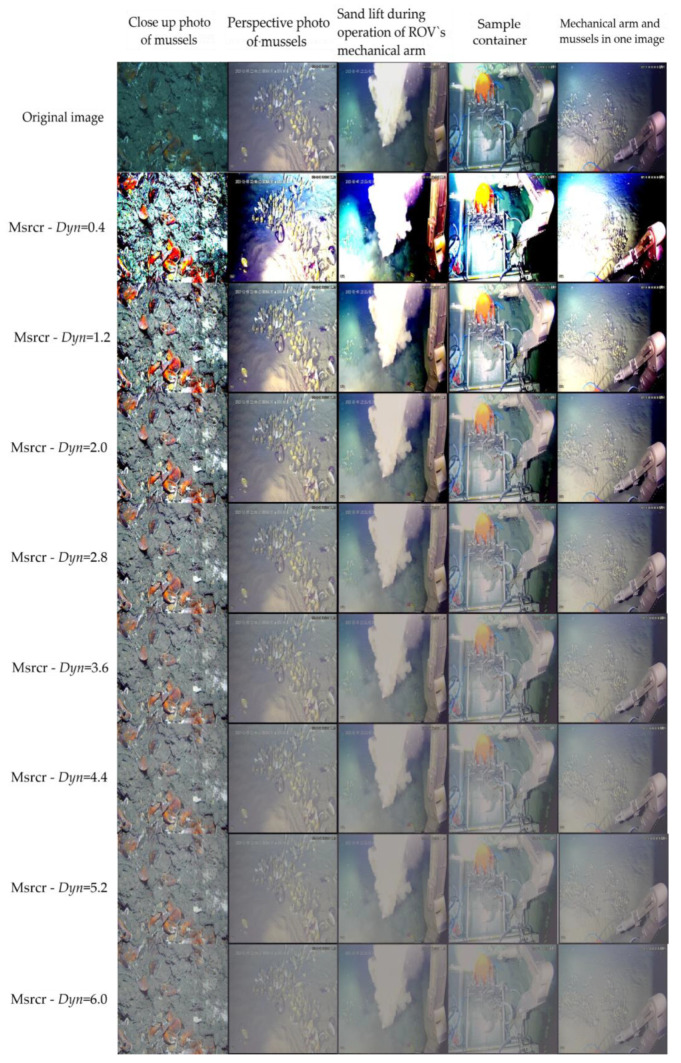
Enhancement of MSRCR under different parameters.

The parameter *Dyn* plays an important role in this method, and according to the representative working conditions in the actual detection of cold seeps with the ROV, we adjusted the relevant parameters for the corresponding images and then modified the relevant parameters of MSRCR. Figure 4 and Table 1 present the quantitative values of the enhancement effect of each parameter. In Figure 4, the first row shows the original images collected by the ROV, and the other rows depict the enhanced images. To ensure that the parameters have a large variation range and a small enough variation interval, we set the value of Dyn from 0.4 to 6.0 and the interval to 0.2 to enhance the cold seep images under different operating modes. The quality of enhancement was judged by the UIQM introduced in [37]. The training dataset was then chosen automatically based on the principle of maximizing the value of UIQM. For different cameras of the Haima ROV under different working conditions, the *Dyn* parameter of MSRCR was distributed in the selected parameter range listed in Table 1. When *Dyn* was 6.0, the value was too high, leading to deterioration with respect to image enhancement. Therefore, we set its maximum value to 6.0 in the experiment. Similarly, *Dyn* = 0.4 was too low, resulting in deterioration with respect to image enhancement; thus, we set its minimum value to 0.4. To make the density of *Dyn* sufficiently high, we set the step size to 0.2 in the experiment. For the sake of brevity, Table 1 only lists the statistical rule of MSRCR enhancement when the step size is 0.8.

To fairly assess these different parameters, we selected the standard metric UIQM and set the same parameters as observed in [37]. We then assessed the enhancement results of each image in the original dataset under different parameters and identified the one that maximized the UIQM’s value. Finally, the images were inserted into the training dataset as the conversion dataset of the original underwater dataset to train CycleGAN.

After a comparison based on the principle of being able to see every detail in the image and that the color should be as natural as possible, we chose the dynamic parameters that led to the maximum value of UIQM and then inserted them into the training dataset. Although MSRCR can enhance underwater images, different parameters need to be used for different scenarios, which seriously affects its practical application. Therefore, we unified the images with the best MSRCR enhancement effect under different parameters into a dataset for different use scenarios and used the dataset to train the CycleGAN model. A total of 80% of the images were randomly selected for training, and the other 20% were used for verification.

### 3.2. CycleGAN Training and Underwater Image Enhancement

We used the MindSpore framework. Compared with other AI frameworks, such as Tensorflow and PyTorch, MindSpore can remain consistent with the python language style, and its intermediate representation technology ensures efficiency [38]. The parameters referenced in Ref. [6] were used for model training. On this basis, to make full use of hardware resources and improve image resolution, we set the image’s resolution to 512 × 512.

All tests were run on a Lenovo P920 workstation. The operating system was CentOS Linux8.6, and the deep learning framework was MindSpore 1.9.0. Using the method proposed in this paper, the total number of images in the dataset was set as 2438 images, corresponding to the five representative underwater working conditions in Figure 4. The CPU used was Intel Xeon 5218R, and the GPU was NVIDIA 3090. All the data were stored on a Lexar NM800 hard disk.

**Figure 5:**
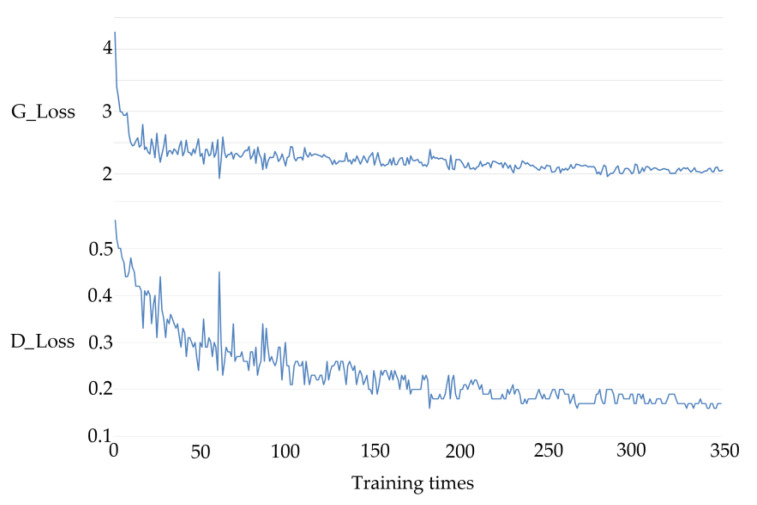
Training loss for CycleGAN.

The curves reflecting the training cycle’s consistency loss (G_loss and D_loss) are shown in Figure 5. In the first 100 iterations, the loss curves descended rapidly, and Table 1 confirms this viewpoint. In the first 100 epochs of training, the UIQM values of images enhanced by the trained CycleGAN model were not stable enough because the variance value was too large; however, after 100 epochs, the mean and variance of the images in the cold seep dataset tended to be stable. The average time cost of training was 17’56” per epoch, so all training processes cost ~105 h for the 350 epochs. Because the loss was reduced very slowly after 200 epochs, the parameters of CycleGAN could be obtained by comprehensively considering the time cost factor of training.

**Figure 6:**
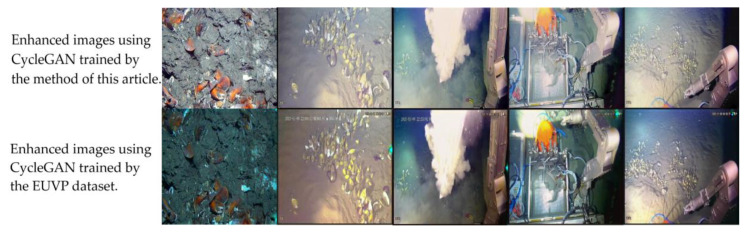
Enhancement results for the trained CycleGAN.

Figure 6 shows the effect of using the trained CycleGAN to enhance several working conditions that were opposite to those indicated in the first line of Figure 4. It can be observed from the figure that the images under all working conditions were brighter, with richer colors and better color consistency after enhancement. As a comparison, the image in the second line is the enhancement effect of the network trained with the standard EUVP dataset (http://irvlab.cs.umn.edu/resources/euvp-dataset accessed on 22 September 2022). It can be observed from the figures that although the images enhanced by CycleGAN, which was trained with the EUVP dataset, seem more colorful, CycleGAN failed in a specific scene (the figure on the left).

**Figure 7:**
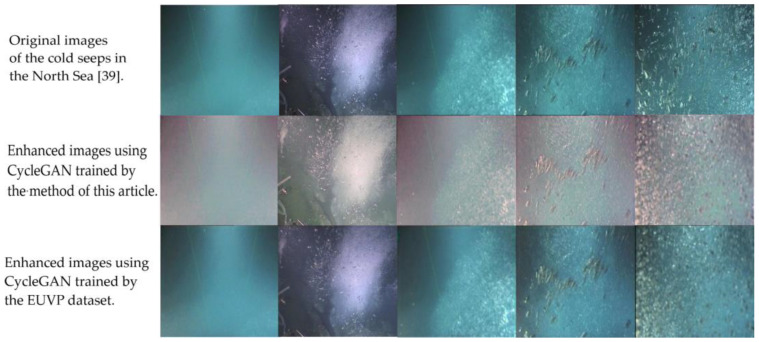
Results for the trained CycleGAN’s enhancement of images in the North Sea [39].

Figure 7 shows the chosen images of the cold seeps in the North Sea [39]. The statistical regularities of the UIQM value for MSRCR and the training steps of CycleGAN are provided in Table 1. From the images and the UIQM values, we can see that, before enhancement, the images’ color was dim, and the target object was not clear. After enhancement, the image color was rich, and the contrast was obvious. Moreover, as with the dataset tests, the UIQM value was still better than that of MSRCR. As seen in the third line of Figure 7, the CycleGAN trained with the EUVP dataset failed to enhance the images collected from cold seeps in the North Sea [39].

### 3.3. Versatility Test of the CycleGAN

**Figure 8:**
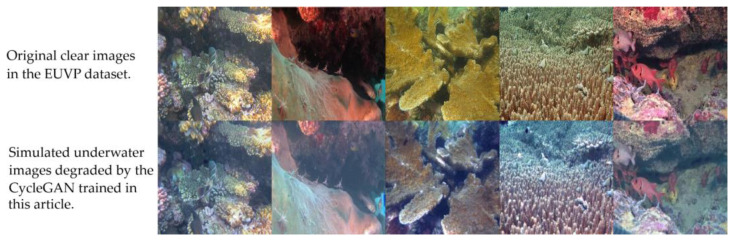
Degradation of enhancement results.

Training the classification model or the target recognition model requires a large number of labeled data. However, in practical applications, obtaining a substantial number of underwater data directly is difficult. Using CycleGAN to degrade images may provide another possibility for training the underwater depth model. As shown in Figure 8, bright images could deteriorate underwater images.

### 3.4. EnlightenGAN Test

To verify the generality of the method proposed in this article, we tested it using EnlightenGAN [32]. The hardware used in the training process was the same as that used for CycleGAN. The AI framework was Pytorch 1.12.1. We made very little changes to the source code shared on Github to adapt the new version of Pytorch. The test took 4 h and 17’56” to finish 200 training epochs. The training efficiency was significantly improved compared with that of CycleGAN. Table 2 shows the statistical UIQM values of each training time for EnlightenGAN. The table indicates that EnlightenGAN can achieve a good result after very little training. The UIQM statistical value reached the maximum at 120 iterations, and further training did not improve the enhancement effect.

**Figure 9:**
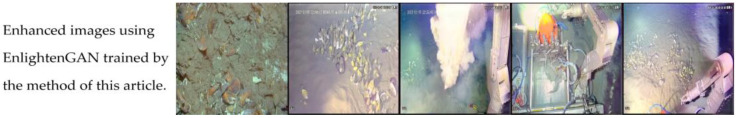
Enhanced images using EnlightenGAN.

Figure 9 shows the images corresponding to Figure 4 and Figure 6. EnlightenGAN parameters of 120 training epochs were selected to enhance the images. It can be seen from the figure that the underwater images were effectively enhanced. The experiment proves this method is also applicable for EnlightenGAN.

## 4. Discussion

By adjusting the parameters of MSRCR, enhanced images with different levels of contrast and sharpness can be obtained. However, a more general scheme is often needed in practical work to reduce the time cost of on-site debugging. In this sense, the CycleGAN trained in this study has obvious advantages because the quality of enhanced images was stable for a variety of scenes in the cold seep area of Haima. We also tested the ability of CycleGAN to degrade the image. The results reveal that the model performed well with the blue background of the sea, whereas brighter images were obviously degraded. Generally, CycleGAN provides a stable degradation output, offering a dataset for the training of underwater image classification models or a target recognition model.

Although a robust CycleGAN model that can be applied to detect cold seep areas can be trained using the method in this study, the contrast of the image enhanced by the trained model still needs to be improved. In addition, more efficient network implementation needs to be considered for high-definition, real-time video stream processing.

The parameters of MSRCR need to be adjusted by conducting experiments to obtain good enhancement effects. Thus, in this study, we creatively employed MSRCR to build a dataset to train CycleGAN. An image enhancement CycleGAN model suitable for a variety of different scenes in the cold seep area of Haima was then obtained. Using the relevant interfaces provided by the MindSpore architecture, the model can easily be deployed in an ROV or in a shipboard computer to improve underwater video quality. 

## 5. Conclusions and Future Steps

In this article, we found a new method for building a dataset with the help of MSRCR and UIQM. Using the dataset, CycleGAN was trained to enhance the images collected from the Haima cold seeps, with good effects. Compared with the CycleGAN trained with the public dataset, the model that we trained can be widely used to enhance images of cold seeps. It will be helpful for the detection of cold seeps in deep seas.

We were effectively able to implement an easy-to-expand dataset-building method to provide effective solutions for underwater image enhancements in different environments.

There are still some shortcomings in this article. The training effect is dependent on the dataset itself, and CycleGAN could not be trained to obtain a larger statistical UIQM value than the dataset we built. In this paper, only the classical CycleGAN was used for experiments. Although this model has been widely used, compared with the newly proposed network, its training efficiency and accuracy are relatively limited. Thus, it still requires some improvements, which could be achieved in future studies, such as the following:

- A better conventional underwater image enhancement method can be found compared to MSRCR, and a new dataset for training can be built.
- Improved datasets can be obtained by implementing better enhancement evaluation methods.
- Our method is generic, and in future work, we will apply more updated models to explore the path of underwater image enhancement and explore more dataset-building schemes.

## Figures and Tables

**Table 1:** MSRCR and CycleGAN image enhancement statistical data of UIQM values for Qiongdongnan and North Sea cold seeps.

MSRCR		**Qiongdongnan Cold Seeps**		**North Sea Cold Seeps**	
	Msrcr *Dyn* values	Mean	Variance	Mean	Variance
	Original images	2.180	0.204	2.454	0.382
	Msrcr-0.4	1.034	0.047	1.488	0.110
	Msrcr-1.2	2.054	0.1632	2.143	0.233
	Msrcr-2.0	2.045	0.219	1.813	0.247
	Msrcr-2.8	1.923	0.234	1.567	0.244
	Msrcr-3.6	1.752	0.236	1.520	0.200
	Msrcr-4.4	1.586	0.228	1.337	0.164
	Msrcr-5.2	1.440	0.213	1.186	0.135
	Msrcr-6.0	1.318	0.197	1.063	0.110
	Msrcr best value chosen	2.444	0.189	2.572	0.197
CycleGAN	CycleGAN Training times	Mean	Variance	Mean	Variance
	20	2.344	0.202	2.572	0.126
	40	2.398	0.181	2.606	0.140
	60	2.478	0.157	2.590	0.171
	80	2.421	0.158	2.572	0.208
	100	2.343	0.171	2.544	0.218
	120	2.489	0.174	2.637	0.215
	140	2.422	0.166	2.628	0.187
	160	2.499	0.154	2.839	0.112
	180	2.465	0.173	2.635	0.234
	200	2.426	0.177	2.647	0.260

**Table 2:** The statistical UIQM values of each training time for EnlightenGAN.

EnlightenGAN Training Times	Mean	Variance
20	2.355	0.190
40	2.384	0.165
60	2.352	0.174
80	2.199	0.177
100	2.231	0.178
120	2.500	0.174
140	2.213	0.187
160	2.189	0.192
180	2.186	0.193
200	2.264	0.176

## Data Availability

The MindSpore framework and basic code of CycleGAN can be downloaded from https://www.mindspore.cn/ (accessed on 4 January 2022) and https://gitee.com/mindspore/models (accessed on 4 January 2022), respectively. The basic code of EnlightenGAN can be downloaded from https://github.com/VITA-Group/EnlightenGAN (accessed on 25 January 2023). The generated dataset, trained model, and enhanced image in the current study are not publicly available because they are the property of the project, but they can be made available from the corresponding author upon reasonable request.
